# A Novel Approach to Measuring an Old Construct: Aligning the Conceptualisation and Operationalisation of Cognitive Flexibility

**DOI:** 10.3390/jintelligence12060061

**Published:** 2024-06-11

**Authors:** Jens F. Beckmann, Damian P. Birney, Robert J. Sternberg

**Affiliations:** 1 School of Education, Durham University, Durham DH1 1TA, UK; 2 School of Psychology, University of Sydney, Camperdown, NSW 2006, Australia; damian.birney@sydney.edu.au; 3 Department of Psychology, College of Human Ecology, Cornell University, Ithaca, NY 14853, USA; robert.sternberg@cornell.edu

**Keywords:** cognitive flexibility, novelty processing, dynamic testing, inhibition, intra-individual variability

## Abstract

A successful adjustment to dynamic changes in one’s environment requires contingent adaptive behaviour. Such behaviour is underpinned by cognitive flexibility, which conceptually is part of fluid intelligence. We argue, however, that conventional approaches to measuring fluid intelligence are insufficient in capturing cognitive flexibility. We address the discrepancy between conceptualisation and operationalisation by introducing two newly developed tasks that aim at capturing within-person processes of dealing with novelty. In an exploratory proof-of-concept study, the two flexibility tasks were administered to 307 university students, together with a battery of conventional measures of fluid intelligence. Participants also provided information about their Grade Point Averages obtained in high school and in their first year at university. We tested (1) whether an experimental manipulation of a requirement for cognitive inhibition resulted in systematic differences in difficulty, (2) whether these complexity differences reflect psychometrically differentiable effects, and (3) whether these newly developed flexibility tasks show incremental value in predicting success in the transition from high school to university over conventional operationalisations of fluid intelligence. Our findings support the notion that cognitive flexibility, when conceptualised and operationalised as individual differences in within-person processes of dealing with novelty, more appropriately reflects the dynamics of individuals’ behaviour when attempting to cope with changing demands.

## 1. Introduction

Intelligence research conducted for well over 100 years has produced a substantial body of evidence for the predictive utility of intelligence tests, be it in terms of predicting success in education (Brown et al. 2021; Richardson et al. 2012; Roth et al. 2015), in vocations (Ree et al. 1994; Schmidt and Hunter 2004; Viswesvaran et al. 2005), in health outcomes (Deary et al. 2021; Der et al. 2009; Gottfredson and Deary 2004), or success in life in general (Deary et al. 2005; Gottfredson 1997; Schmidt 2014). This broad spectrum of evidence represents a core element in the narrative of the success story of intelligence testing.

There is, however, a tendency to interpret this success in predicting various kinds of achievements and accomplishments as some form of validity. This tendency, we will argue, creates the risk of stalling progress in conceptual work towards understanding intelligence and, ultimately, in developing even more useful assessment tools. Equating predictive utility with validity tends to mask unresolved tensions between the conceptualisation and operationalisation of intelligence as a cognitive ability construct. That is, by pragmatically contenting oneself with the utility of test scores in predicting various quantifiable indicators of societally sanctioned success, one risks diverting attention from the cardinal question of validity, that is, whether test scores are in fact manifest indicators of the construct—in this case, intelligence—as it is conceptually defined.

This paper aims to contribute to and move forward the debate around the discrepancy between the conceptualisation and operationalisation of cognitive abilities by seizing the opportunities afforded by the revitalised interest in what can tentatively be described as dynamic aspects of cognitive abilities (see Sternberg 2019, as an example). To that effect, we present a *conceptually informed proof-of-concept study*. This requires us to first deliberate on potential conceptual links between cognitive flexibility (CF) and intelligence. Subsequently, we will discuss the extent to which these links are, or are not, reflected in the operationalisation of intelligence. We then present an empirical study that tests some of the conceptual arguments made here, before we conclude with the contention that progress in measuring intelligence as a dynamic construct of cognitive abilities—which refers to the construct of cognitive flexibility—must be based on conceptual work, rather than relying on (post hoc) interpretations of patterns in test intercorrelations.

## 2. Conceptual Foundation

### 2.1. Cognitive Flexibility—A New Name for an Old Thing?

*To invent without scruple a new principle to every new phenomenon, instead of adapting it to the old; to overload our hypotheses with a variety of this kind; are certain proofs, that none of these principles is the just one, and that we only desire, by a number of falsehoods, to cover our ignorance of the truth*.

*(Hume, 1739–40)*

The history of intelligence research provides many examples of what might be mistaken as “productivity in creating”, or more appositely, the *construction* of new intelligence constructs, be they multiple (Gardner 1983), emotional (Salovey and Mayer 1990), cultural (Earley and Ang 2003), sexual (Klein 2012), practical (Sternberg et al. 2000), affective (Marcus et al. 2000), operational (Dörner 1986), or adaptive (Sternberg 2019). A potential benefit and allurement of such “innovativeness” is that a new construct label comes with the promise of relative freedom from some of the reputational baggage that already established labels might carry. As most would agree, the concept of “intelligence” has had its fair share of such baggage (Gottfredson 2018). But the promise of such a starting afresh tends to also come with unintended side effects. These include the risk of contributing to an inflationary use of the concept, which tends to diminish its explanatory value and utility. The prize of attempting to overcome identified shortcomings of “the old and traditional” can come at the cost of introducing new limitations. Concerns such as these are a fixture in the long and productive history of intelligence research. McNemar’s witticism “… the first cardinal Principle of Psychological Progress: *Give new names to old things*” (McNemar 1964, p. 872, italics in original) is only one of many reminders that critical reflections of this sort are anything but new. David Hume’s admonition, quoted above, provides only cold comfort that such tendencies seemed to have been of concern far before psychology’s attempts to establish itself as a scientific discipline, and are therefore not limited to psychological research.

Our focus is on cognitive flexibility, and we ask, is it just a new name for an old thing? The answer to this question from a *conceptual* angle will be, yes. The answer from an *operational* angle, however, will have to be, no. As a first step in elaborating this conundrum and to subsequently proposing how to resolve it, we attempt a conceptual specification of the construct of cognitive flexibility.

### 2.2. Defining Cognitive Flexibility

Cognitive flexibility as a construct has been discussed as an ability of various kinds, as an aptitude, a capacity, an attitude, etc. Conceptualisations of cognitive flexibility include the *abilities* to shift across concepts and situations (Chi 1997), to change one’s mindset (Frensch and Sternberg 1989), to adjust to changing demands (Lezak et al. 2012; Scott 1962), or to switch modes of response (Kossowska et al. 1996). Cognitive flexibility has also been discussed as an *aptitude* (for changing lines of thinking, Garaigordobil 2006) and as a *capacity* (for adaptive functioning, Hund and Plumert 2005). Others have conceptualised flexibility as a conative disposition or *attitude* (Luchins and Luchins 1959), or in terms of awareness, willingness, and self-efficacy (Martin and Rubin 1995). Wand (1958) suggested “… to relinquish the concept flexibility as an ability and to conceive of it as a manner of approach to a problem affecting the capacity for new learning” (p. iii).

In an attempt to reconcile these different perspectives and the risk of conceptual ambiguity (ability, aptitude, attitude, capacity, etc.), (Yu et al. 2019) recently proposed cognitive flexibility to be considered a meta-competency. The use of the concept *competence* has advantages, but it also brings considerable disadvantages. One of the pragmatic advantages is that declaring something a competency immunises it from potential refutation (i.e., one tends to be always not wrong). Also, as performance-oriented behaviours in real-life contexts cannot be meaningfully described under a lens of conceptual purity, competency measures tend to provide promising levels of predictive utility of such behavioural outcomes. This, at the same time, constitutes a limitation, because predictive utility, as discussed earlier (and elsewhere, Birney et al. 2022), cannot substitute for evidence of (construct) validity. Another limitation of settling for competency as the conceptual description of cognitive flexibility is that it questions the meaningfulness of any within- or between person comparisons. As competencies are a conglomerate or amalgamation of knowledge, attitude, affect, cognitive abilities, and other constructs, it is likely that this mixture is different for different individuals (despite comparable performance), and it might also be different across time as a result of intra-individual change processes (e.g., learning). This dynamic multidimensionality challenges the assumption that necessarily underpins scale aggregation and subsequent inter- and intra-individual comparisons, namely, to have measured a construct on a unitary scale.

#### 2.2.1. Cognitive Flexibility as Executive Function

By reviewing the considerable body of research literature related to cognitive flexibility (e.g., Yu et al. 2019), it becomes apparent that our understanding and definitions of cognitive flexibility are strongly influenced by the tasks used to measure the construct. In the neuroscience literature, cognitive flexibility is discussed as an emergent property of efficient executive function (Banich 2009; Dajani and Uddin 2015; Diamond 2013). One of the three core executive functions in Diamond et al.’s framework is cognitive flexibility. It refers to the ability to change perspectives, which requires inhibition of (or disengagement from) previously taken perspectives. It also incorporates processes of set shifting (Yerys et al. 2015) and task switching (Monsell 2003). Prominent tools used to tap into these processes include the Trail-Making Test (TMT-B, Arbuthnott and Frank 2000), the Wisconsin Card-Sorting Task (WCST, Grant and Berg 1948), and the Stroop Task (Kalanthroff et al. 2018; Stroop 1935).

In his unity–diversity theory of executive function, Miyake (e.g., Miyake et al. 2000) discusses three factors—updating, shifting, and inhibition. Updating refers to processes of monitoring and updating information in terms of their relevance to the task at hand. Shifting refers to processes of disengaging from irrelevant and engaging in relevant mental sets or operations in response to changes in task and situation demands. Inhibition refers to processes of actively and deliberately overriding automatised or prepotent response patterns when necessary. Processes of updating, shifting, and inhibition are functionally interrelated, structurally separable, and they show a differential predictive utility of performance in complex executive function tasks such as the ‘Tower of Hanoi’ task (ToH, Welsh 1991). Inhibition is considered to serve as the unifying element (i.e., what is common across executive functions), whilst updating and shifting represent the elements contributing to diversity in Miyake’s unity–diversity theory of executive function (Friedman et al. 2006).

It is important to note, however, that mere references to tasks used to nominally measure cognitive flexibility, including tests of executive function, cannot answer the requirement for a conceptual definition. In borrowing from Boring (Boring 1923, June 6), an operational definition (i.e., when conceptualisation follows operationalisation) would state that cognitive flexibility is what the WCST (or similar) measures. (Frick et al. 1959) provide an early example of an attempt to define cognitive flexibility *psychometrically* by factor-analysing covariances amongst 28 experimental tasks and reference tests for cognitive abilities, many of which are part of various standard measures of intelligence. They identified two flexibility factors, *spontaneous flexibility* and *adaptive flexibility*. The tasks affiliated with the factor labelled “spontaneous flexibility” (containing the “unusual uses” task, for example) share the demand for producing a diversity of ideas in a relatively unstructured situation. The tasks that constituted the “adaptive flexibility”-labelled factor (containing “insight puzzles”, for example) share the demand for a set change (or a change of reference) in order to meet requirements imposed by changing problems. As is the case with any *formative* measure of psychological constructs, a definition of cognitive flexibility based on such approaches tends to depend on the selection of tasks used (and the sample studied) in its factor-analytic formation.

In contrast, cognitive approaches tend to start with a conceptualisation that then informs the design of tasks that serve the purpose of being *reflective* measurements of the targeted construct. In the case of cognitive flexibility, this means that its operationalisation is based on theories of information processing. Whilst this already hints at conceptual relatedness, if not overlap with fluid intelligence, the specific emphasis of such a theorisation of cognitive flexibility is on the processing of novelty. Processing information that is sufficiently novel in relation to existing experiences, knowledge, or routines requires strategic responses to ambiguity and unfamiliarity in dynamic environments (e.g., Cañas et al. 2003) and is typically characterised as adaptive performance. From the perspective of cognitive approaches, the answer to the question of whether cognitive flexibility is a new, or at least a sufficiently distinguishable, construct requires reflection regarding whether (and if so, to what extent) cognitive processes involved in processing familiar information differ from those involved in processing *novel or unfamiliar* information.

#### 2.2.2. Cognitive Flexibility as Intelligence

Based on the definitory reflections outlined so far, it is already becoming clear that, conceptually, cognitive flexibility is nothing new or different from intelligence. In fact, and rather unsurprisingly, cognitive flexibility has always been part of definitions of intelligence. For an early example, the concept of cognitive flexibility finds its representation already in William Stern’s definition of intelligence as “… the general capacity of an individual consciously to adjust his thinking to new requirements” and its characterisation as a “… general mental adaptability to new problems and conditions of life” (Stern 1914, p. 3). In the context of Spearman’s proposal of “ultimate laws that govern all cognition” (Spearman 1927, p. 162), intelligence has been discussed as “conscious adaptability to new situations”. These considerations also include reflections on whether “*g*” is a measure of immediate or progressive adaptability. The former is taken to signify success in the first attempt at a new task, the latter to reflect eventual success after prolonged practice^1^ (Spearman 1927, p. 88). Cognitive flexibility also finds its representation in Guilford’s (Guilford 1956) structure of intellect framework, through the operations of divergent production across products and contents. Cattell’s definition of fluid intelligence (Cattell 1971) as the ability to solve novel problems also seems close to what might be considered cognitive flexibility. In fact, fluid intelligence has been discussed as a prerequisite for solving novel problems and using adaptive problem-solving strategies in reasoning, and as essential in coping with unfamiliar situations.

For some, notions of flexibility also coincide with notions of malleability. Although they are conceptually different, studies of the trainability of intelligence (which are far from new; see, for instance, (Stankov 1986) for a summary of the training of flexibility of thought in schools by Radivoy Kvashchev in the early 1960s) and concepts of plasticity and flexibility (Lövdén et al. 2010) are premised on an understanding of underlying dynamic processes.

Another example of the notion that cognitive flexibility has a long tradition of being a core element of most conceptualisations of intelligence includes its representation as the ability to deal with novelty in the experiential sub-theory of Sternberg’s triarchic theory of intelligence (e.g., Sternberg 1984). In reference to more contemporary conceptualisations of intelligence, within the Cattell–Horn–Carroll (CHC) model of cognitive abilities, fluid reasoning (Gf)—as one of the broad factors therein—is defined as “deliberate but flexible control of attention to solve novel ‘on the spot’ problems that cannot be performed by relying exclusively on previously learned habits, schemas, and scripts” (Schneider and McGrew 2018, p. 93). To summarise so far, *conceptually*, at least, cognitive flexibility appears to be an old name for an old thing.

### 2.3. Problem, What Problem?

Logic would now seem to dictate that if cognitive flexibility is assumed to be an essential facet of our conceptual understanding of intelligence, and given the relative success of intelligence testing in predicting a wide range of practically relevant life outcomes, then intelligence tests should also be considered valid assessment tools for measuring cognitive flexibility. There are, however, at least two flaws in this logic. The first flaw has been highlighted earlier, namely, interpreting predictive utility as (construct) validity, which the above inference would imply (for some further elaboration on this point, see Borsboom and Mellenbergh 2007; Borsboom et al. 2009). The second flaw becomes apparent when one realises that the cognitive demands posed by items in conventual tests of cognitive abilities tend not to require one to change their mindset, to switch modes of response, to adjust to changing demands, or to break out of routine ways of thinking when necessary (Matthew et al. 2008). In short, the lack of opportunities for test takers to exhibit behaviour that is indicative of their ability to deal with novelty results in an underrepresentation of the target construct in test scores. Such construct underrepresentation (Messick 1989) suggests a serious validity problem, which, however, tends to be masked by the success of the tools we use in predicting more or less readily available quantifications of a multitude of achievements and pertinent performances.

This brings us back to the initially asked question, that is, whether cognitive flexibility is a new construct that needs special attention. Addressing this question from a *conceptual* angle, cognitive flexibility and intelligence show a substantial overlap with one another, suggesting that cognitive flexibility is anything but a novel construct. However, considerations from an *operational* angle reveal that conventional approaches to measuring intelligence are unlikely to be adequately valid indicators of individual differences in cognitive flexibility, which in turn suggests that cognitive flexibility is something new, albeit operational rather than conceptual, and therefore demands our attention.

### 2.4. Redressing the Discrepancy between Conceptualisation and Operationalisation

How can this conundrum, which originates from a discrepancy between conceptualisation (i.e., how we define the target construct) and operationalisation (i.e., how we measure that target construct), be resolved?

Two options seem to be available. The first option would be to adjust and refine the operationalisation, so that test scores derived from tests of (general) cognitive abilities are reflective of individual differences in the ability to deal with novelty. The second option for addressing the discrepancy between conceptualisation and operationalisation would be to resemble an operational, data-driven approach, by which a “theory” is adjusted to reflect the data obtained using the conventional operationalisation. We pursue the first option. To that end, we aim to incorporate opportunities in the testing procedure that enable test takers to demonstrate their ability to deal with novelty, to adjust to changes, and to break out of routine ways of thinking when necessary.

#### 2.4.1. The Concept of Novelty

Cognitive flexibility is required when one is confronted with incongruent information. Such incongruency can appear in two different constellations. The first is likely to induce the challenge of *ostensible novelty*. This challenge occurs in situations that tend to give a false impression of being unfamiliar and novel, whereas the skills, knowledge, experiences, or competencies one has already acquired would suffice to tackle the task at hand. The second form of incongruency occurs when a situation is falsely perceived as being familiar, whereas tackling it actually would require different or novel approaches. This form of incongruency creates the challenge of *obscured novelty*. (Beckmann 2014) has previously introduced the notion of “the three Os of novelty”, where the third form (in addition to *Ostensible* and *Obscured*) represents what one might label as *hOnest novelty*. That is novelty where situational cues suggest unfamiliarity, and the challenge is to identify the novel approaches necessary to solve the problem at hand. One could argue that dealing with this kind of novelty is covered by traditional approaches to measuring fluid intelligence, or in research paradigms related to “classical” problem solving. Our conceptual focus with cognitive flexibility is primarily on dealing with ostensible novelty and obscured novelty.

Overall, cognitive flexibility manifests itself in the ability to tell the two facets of novelty apart and to act upon them accordingly. This renders cognitive flexibility as an enabler for behaviour that responds to adaptive contingencies (Birney and Beckmann 2022)—one’s adaptivity is reflected in functional changes in behaviour that are contingent on the task and situational dynamics of the environment, concordant with one’s capabilities to respond.

#### 2.4.2. Operationalising Cognitive Flexibility

To inform a construct-adequate operationalisation of cognitive flexibility, we draw on a conceptual framework comprising the person, the task, and the situation (PTS framework) (Beckmann 2010; Birney et al. 2016; Beckmann et al. 2017; Tromp and Sternberg 2022). Whilst the *person* dimension subsumes psychological attributes such as reasoning ability or cognitive flexibility (to mention only two characteristics), the *task* dimension comprises two sub-facets. These are the *task qua task* and the ‘task as behaviour requirement’ (Hackman 1969; McGrath and Altman 1966; Wood 1986). The ‘task qua task’ facet refers to the physical, and therefore perception-relevant, characteristics of the stimuli a test taker might be confronted with. The *task as behaviour requirement* refers to what the test taker is instructed to do (e.g., to complete an analogy). Both facets contribute to the complexity of the task in terms of the cognitive work that is to be executed to tackle it. The *situation* dimension refers to the circumstances under which a task is to be performed (akin to the notion of “task environment” as described by (Newell and Simon 1972, p. 55). This includes, for instance, whether the task is to be performed under time pressure, or what answer format is utilised in the task. The situation facet also contributes independently from the task dimension to the overall complexity (of the task–situation combination). Individuals differ in their capabilities to deal with this overall complexity, which manifests itself in the differing degrees of difficulty they experience whilst performing the given task(s). Differences in experienced levels of difficulty are operationalised as test scores.

The distinction between the task and the situation—as one of the core features of the PTS framework—is of particular importance. This distinction tends to be often overlooked, mainly because tasks—that is, specified requirements for cognitive behaviour in a given context—tend to be always performed, analysed, and interpreted in standardised situations. For instance, controlling for situation-related variance across a set of tasks is essential for being able to interpret observed variance in performance scores as indications of intra-individual differences in several person attributes in the context of psychometric assessments. However, as the same task can be performed under different situational constraints, the task and situation need to be conceptualised as independent contributors to complexity.

We aim to resolve the issue of the discrepancy between conceptualisation and operationalisation in relation to the measurement of flexibility as cognitive ability. We do so by creating a construct-adequate operationalisation that is based on the systematic manipulation of task and situation features to induce different challenges for dealing with novelty in cognitive tests.

To not succumb to a false innovativeness (as referred to earlier in this paper), we make the effort to anchor our novel approach to measuring intelligence as cognitive flexibility in well-established item paradigms. The recourse to established item paradigms that are used in the measurement of reasoning ability reflects a conceptually informed definition of cognitive flexibility as an (albeit underrepresented) facet of intelligence.

In the following, we present designs for two such tasks that utilise analogies and classification problems, respectively.

#### 2.4.3. Cognitive Flexibility when Dealing with Obscured Novelty

**Figure 1:**
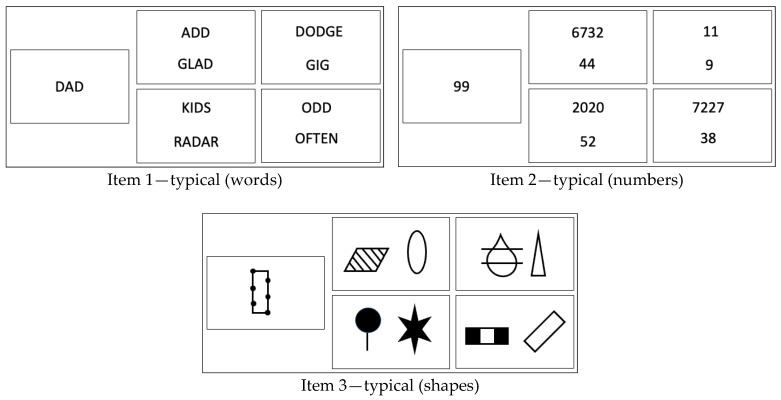
Three examples for domain-typical items in the Flexible Inference Task across three stimulus domains (for solutions, see Appendix A).

The Flexible Inference Task employs classification problems. Test takers are asked in each item to identify the pair of stimuli that best match a target stimulus based on their shared characteristics or features. The stimulus material used in the Flexible Inference Task are numbers, words, and shapes to achieve a domain balance across all items (Figure 1).

**Figure 2:**
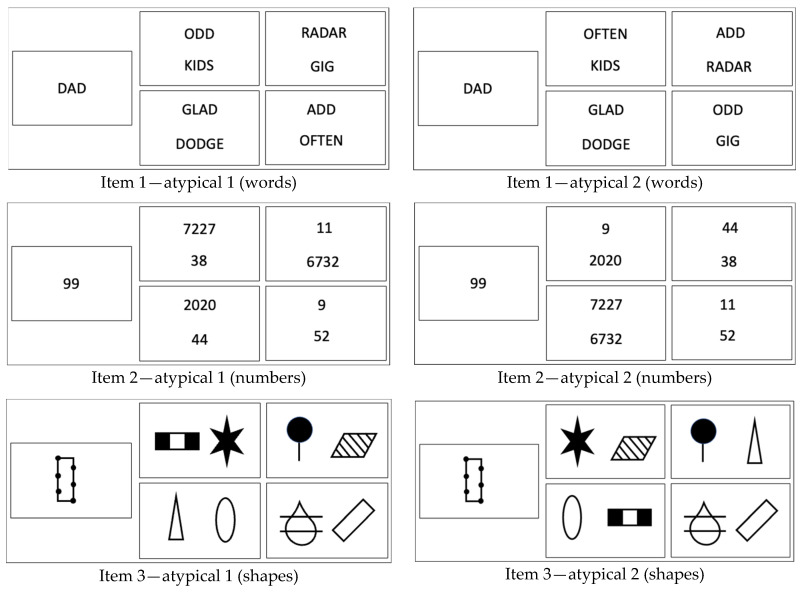
Examples for domain-atypical items in the Flexible Inference Task across three stimulus domains (for solutions, see Appendix A).

To evoke the demand to deal with novelty, the elements of each of the stimulus pairs are rearranged in two subsequent items, forming an item triplet (Figure 2). Within each of these resulting item triplets, the task in both of its sub-facets, that is, the *task qua task* (i.e., the same stimulus material) and *task as behaviour requirement* (i.e., solve the classification task), remains constant. A variation within each item triplet, however, is introduced by changing the topological arrangement of the stimuli. The variation in this situation characteristic, whilst keeping the task constant (see PTS framework), creates a constellation in which the previously induced classification rule does not apply anymore. Dealing with this change requires inhibition of the response that might have been appropriate to the previous items in the triplet with the identical target, and it demands a re-engagement in identifying a new rule, despite familiarity with the set of stimuli. In short, the variation of a situational feature, whilst keeping the task constant, induces the demand to deal with *obscured novelty*.

To summarise, items with the same target and the same—yet rearranged—set of stimuli constitute an item triplet. The first element of each triplet represents a *domain-typical* classification item (i.e., the to-be-inferred classification rule is based on numerical characteristics in items using numbers as the stimulus material, or semantic meanings in items that use words as the stimulus material, see Figure 1). The subsequent rearrangement of elements of each item triplet creates *domain-atypical* classification items (for instance, the to-be-inferred classification rule is based on graphical features of digits in items using numbers as stimuli, or it is based on the number of vowels in items using words as stimuli, see Figure 2 for examples).

It is to be expected that domain-atypical items would be more difficult than the domain-typical counterparts that precede them. In terms of cognitive processing demands, the *unfamiliarity* or *novelty effect* (due to the demand to infer a domain-atypical classification rule) will be complemented by a *transition effect* caused by the requirement to inhibit previously adopted perspectives on the same set of stimuli. More speculatively, we also assume that the transition between the first domain-atypical to the second domain-atypical classification item will be less impactful. This might result in an attenuated novelty effect; it might even result in a slight recuperation or “recovery”. Testing these and other conceptually informed expectations empirically form part of a meaningful validation strategy (Borsboom et al. 2004).

To be successful in the Flexible Inference Task, a flexible use of different frames of reference for otherwise familiar stimuli is necessary. The ability to inhibit experience gained on previous items is the prerequisite for using different cognitive approaches to the same set of stimuli. Generally, we expected that the intra-individual variability in performance scores caused by the systematic variation within each item triplet will be indicative of test takers’ ability to deploy their cognitive resources flexibly.

#### 2.4.4. Cognitive Flexibility when Dealing with Ostensible Novelty

**Figure 3:**
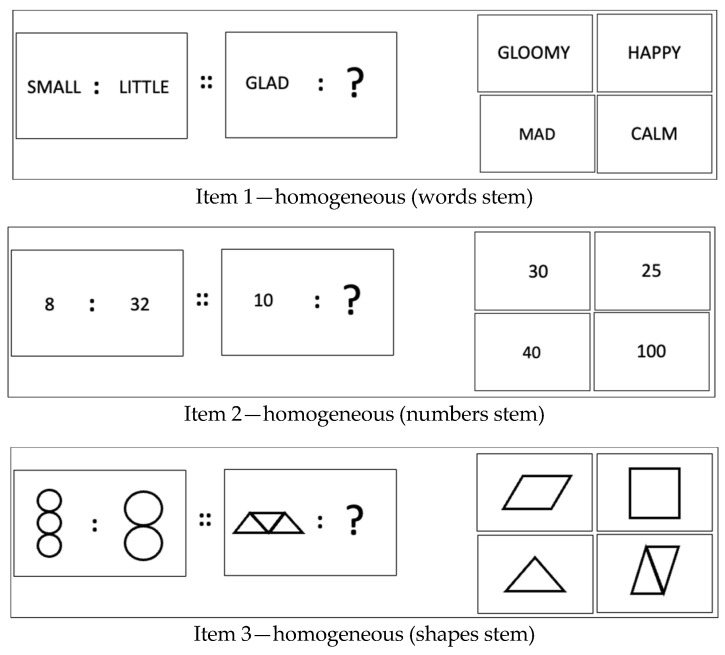
Three examples for domain-homogeneous items in the Flexible Mapping Task across three stimulus domains (for solutions, see Appendix A).

The newly developed Flexible Mapping Task uses analogy items of the type “A is to B as C is to?” The stimulus material used in the Flexible Mapping Task are numbers, words, or shapes to achieve a domain balance across all items (Figure 3).

In terms of a componential analysis of the task requirements to solve analogy problems (e.g., Sternberg 1983) in the Flexible Mapping Tests, test takers are expected to work through a sequence of cognitive processes. These include (1) to encode the characteristics of the terms given in the analogy stem (A and B), (2) to infer the relation between these two terms (A : B), (3) to map this inferred relation to the third term (C), (4) to apply the rule to the third term to produce the missing fourth one (D’), and finally—under the condition of multiple choice—(5) to justify the decision, which answer option completes the analogy according to the rule applied.

**Figure 4:**
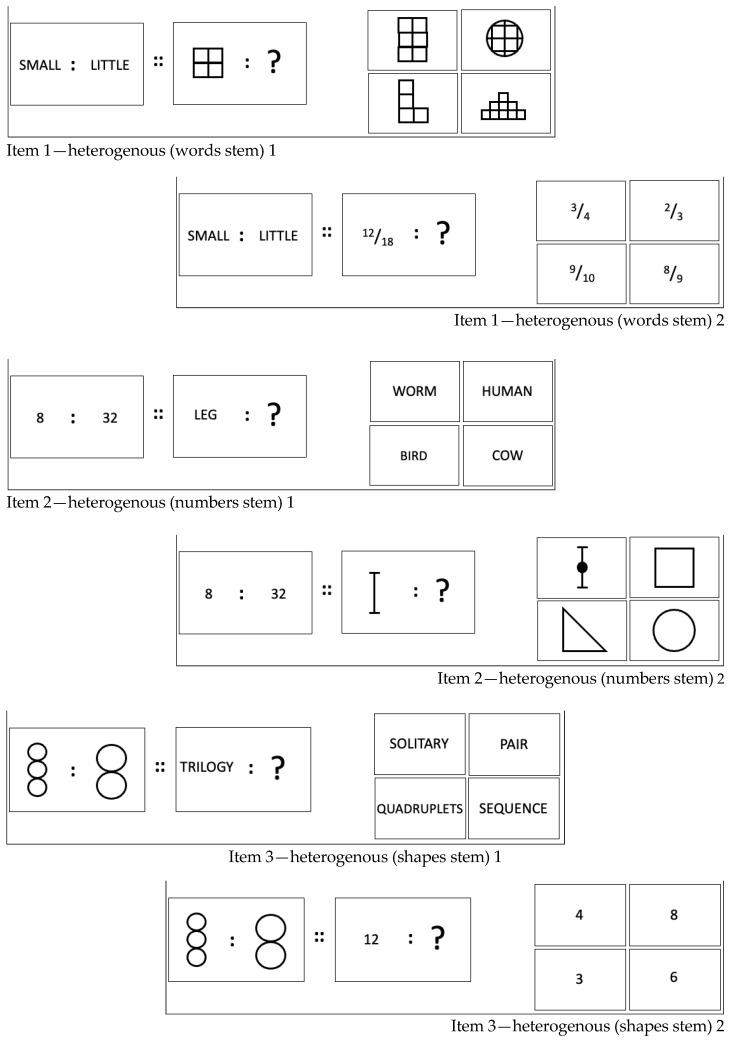
Examples for domain-heterogeneous items in the Flexible Mapping Task across three stimulus domains (for solutions, see Appendix A).

To evoke the demand to deal with novelty, the domain which the terms C and D are from will be different to the domain of the terms A and B (i.e., the analogy stem) in subsequently presented items. This manipulation of a situational feature, whilst holding the task constant, creates so-called domain-heterogeneous items (see Figure 4) in which the demand to map the previously inferred relationship rule across domains is introduced.

As in the Flexible Inference Task, items are presented in item triplets. Each triplet’s first element is a “conventional” domain-homogeneous item (i.e., all terms are either numbers, words, or shapes). The second and third element of each item triplet in the Flexible Mapping Task are domain-heterogeneous, in which the analogy stem remains the same, but the two other terms are from a different domain (Figure 4). Across all item triplets in the Flexible Mapping Task, every domain is combined with every other domain.

Importantly, as the analogy stem remains the same across the three items in each item triplet, the to-be-inferred rule remains the same too. The challenge “merely” is to map (and apply) this very rule onto the other domain. In terms of the PTS framework, the change of a situational feature (e.g., domain heterogeneity), whilst keeping the task constant (i.e., solving the analogy), is intended to create *ostensible novelty*. Being able to resist a false sense of novelty and not to engage in unnecessary steps to infer a new rule, but to focus rather on utilising and applying already established insights, is expected to be a construct-adequate operationalisation of the second facet of cognitive flexibility, that is, the ability to deal with ostensible novelty.

We expect domain-heterogeneous items in the Flexible Mapping Task to be more challenging than their domain-homogeneous equivalents. This difficulty differential is expected to be caused by mapping distances that extend across domain boundaries. Similar to the Flexible Inference Task, we expect to observe a slight performance “recovery” in the third element in each item triplet, despite it being domain-heterogeneous. This expectation is underpinned by the assumption that, after tackling the first domain-heterogeneous instantiation of the analogy, the inferred relationship between the two elements of the analogy stem will be represented on a less domain-specific level of abstraction, which will facilitate its “recycling” when a problem solver is tackling the third element of the respective item triplet.

Whereas the Flexible Inference Task focuses on the ability to infer different relations flexibly (i.e., to deal with *obscured* novelty), the focus in the Flexible Mapping Task is on the ability to map the same rules/relations flexibly (i.e., to deal with *ostensible* novelty).

### 2.5. Aims: Research Objectives

In this exploratory proof-of-concept study, we put some of our conceptually derived assumptions and expectations to the test. With the two newly designed flexibility tasks, we aimed to evoke behaviour that is indicative of an individual’s cognitive flexibility, where cognitive flexibility is conceptualised as defined earlier in this paper. To that end, we used systematic variations of the situational characteristics with which reasoning tasks are presented. In reference to Sternberg’s cognitive components framework (e.g., Sternberg 1988), the sequence of information-processing steps involved in solving conventional classification tasks or analogy tasks comprises ‘encoding’, ‘inference’, ‘mapping’, ‘application’, ‘comparing’, ‘justification’, ‘preparation’, and ‘response’. We argue that the systematic manipulation of situational characteristics realised within each of the item triplets contributes to complexity by adding ‘inhibition’ to the sequence of cognitive processing steps in these tasks.

In the Flexible Inference Task, the flexibility-evoking challenge is to inhibit the tendency to focus on the domain-typical features of the stimuli. For example, one must not inappropriately concentrate on the semantic meaning of the words used in word problems, or search for a calculation-based rule to classify the stimuli in numerical problems. In the Flexible Mapping Task, the flexibility-evoking challenge is to inhibit the tendency to perceive the second (and third) instance of the analogy within a triplet (all with the same analogy stem) as a new analogy. Success in inhibition prevents one’s unnecessary engagement in cognitive processes of ‘re-encoding’ and ‘re-inferring’, as the results of these steps apply across all three items within the respective triplet. Taken together, the added cognitive processing step of inhibition is expected to be reflected in an increase in the difficulty participants experience when solving the domain-atypical classification items in the Flexible Inference Task, or the domain-heterogeneous analogy items in the Flexible Mapping Task, when compared with their performance on their domain-homogeneous or domain-typical counterparts. This expectation constitutes the to-be-tested *Complexity Assumption*.

The second set of assumptions to be tested refers to the question of whether the two flexibility-evoking item categories (i.e., the domain-atypical classification in the Flexible Inference Task and domain-heterogeneous analogies in the Flexible Mapping Task) are psychometrically separable from their conventional counterparts. This constitutes the *Separability Assumption*.

The third and final set of to-be-tested assumptions refers to the question of whether the newly developed flexibility tasks show incremental value in the prediction of a criterion that is reflective of a person’s ability to adjust to changes or to deal with novelty as discussed earlier in this paper. This constitutes the *Predictive Utility Assumption*.

## 3. Materials and Methods

### 3.1. Participants

The study involved *N* = 307 first- or second-year university students studying a diverse range of majors from four universities in the Northeast, and one university in the Northwest, of the USA (*N*_1_ = 88; *N*_2_ = 83; *N*_3_ = 93; *N*_4_ = 43). Participants were recruited through university-wide fliers and e-mail announcements. Participants were paid USD 40 for participation in two 1.5 h testing sessions. A total of 68% of the participants were female. The average age of the participants was 19.6 years (SD = 1.4).

The data collection for this study was part of a larger research project. This resulted in a situation in which not all *N* = 307 participants were presented with the same subset of tasks or tests. In Table 1, we report the actual numbers of complete data sets that were available for each of the respective analyses.

### 3.2. Measures

#### 3.2.1. Flexible Inference Task

For the measurement of the ability to deal with obscured novelty as one facet of cognitive flexibility, we created a total of 45 triplets of classification items for the Flexible Inference Task (for examples, see Figure 1 and Figure 2). Within each of the item triplets, the solution for one item was based on a domain-typical classification rule, whereas for the other two items of that triplet, the classification rule referred to domain-atypical characteristics of the same yet re-arranged set of stimuli.

Any given participant was presented with 5 item triplets from a randomly selected domain (e.g., numbers), followed by 5 item triplets from another domain (e.g., shapes), to be finally presented with 5 item triplets from the remaining domain (e.g., words). Overall, there were 6 possible orders of domains. Whilst item triplets were grouped by domain, the order in which items within each item triplet were presented was randomly selected from 6 possible permutations. That is, participants were randomly allocated to conditions in which the domain-typical items were presented first (followed by the two domain-atypical items), or second (flanked by the two domain-atypical items), or third and last (i.e., preceded by the two domain-atypical items). In short, 6 different item orders were realised. Participants were randomly assigned to one of the three item groups. In this way, we achieved a balance across stimulus domains, whilst at the same time presenting participants with a reasonable number of items, so to help to balance the risk of fatigue with the chance to obtain sufficiently trustworthy estimates of performance. This design aimed to control for potential order effects, be it at item, item triplet, or domain level, in order to enable a robust test of the three assumptions outlined above.

After each response, we provided accuracy feedback, together with an indication of the correct answer option and a concise rationale for it. This feature was aimed at creating a demand for the inhibition of the rule inferred (and/or ultimately explained) that was relevant for the preceding item within the respective item triplet.

#### 3.2.2. Flexible Mapping Task

For the measurement of the ability to deal with ostensible novelty as a second facet of cognitive flexibility, we created a total of 45 triplets of analogy items for the Flexible Mapping Task (for examples, see Figure 3 and Figure 4). Within each triplet, there was one domain-homogeneous item, that is, all elements of the analogy were from the same domain (e.g., all words). The remaining two items within each triplet were domain-heterogeneous, that is, the stimulus domain of the third and fourth term in the analogy differed from the domain of the first and second elements (i.e., the analogy stem).

As is the case for analogy items of the type A : B :: C : ?, the inferred relationship between A and B needed to be mapped onto C in order to identify the analogy-completing term D. The requirement to deal with ostensible novelty was created by the demand to map the relationship onto elements from a domain that differed from the one represented in the analogy stem. In other words, domain-heterogeneous analogy items required bridging a wider mapping distance than their domain-homogeneous counterparts. The ability to flexibly bridge different mapping distances caused by introducing the domain heterogeneity of the stimuli used in the analogy was conceptualised as the ability to deal with ostensible novelty.

In the Flexible Mapping Task, any given participant was presented with 6 item triplets from a randomly selected domain^2^ (e.g., words), followed by 6 item triplets from another domain (e.g., numbers), to be finally presented with 6 item triplets from the remaining domain (e.g., shapes). Overall, there were 6 possible orders of domains to which participants were randomly allocated. For the Flexible Mapping Task, two different presentation modes were realised. When presented in *sequential mode* (S), items within each triplet were presented sequentially on individual screens. When presented in *cumulative mode* (C), items of a triplet were presented cumulatively, that is, the previous item from the respective triplet remained on the screen during the presentation of the subsequent element of that triplet. Within each domain group of 6 item triplets, the first 3 item triplets were presented in either the sequential mode or the cumulative mode. The second set of 3 item triplets within each domain group were then presented in the mode alternative to the first set of the three item triplets. The order of the presentation mode was counterbalanced by randomly allocating participants to either the [Sequential—Cumulative], or the [Cumulative—Sequential] condition. There were 2 possible orders in which items within each item triplet were presented (e.g., [homogeneous, heterogeneous1, heterogeneous2] or [homogeneous, heterogeneous2, heterogeneous1]). All the above randomisations were realised for three different groups of item triplets ([T1 … T5], [T6 … T10], [T11 … T15]). To accommodate the balancing of the presentation mode, which resulted in the necessity to have 6 triplets per domain, the 5 item triplets per domain from each group were supplemented by a randomly selected item triplet from one of the other two item groups. By controlling for potential effects of the presentation mode and various order effects, this design enabled a robust testing of the three assumptions outlined above.

As was the case for the procedure for the Flexible Inference Task, after each response to the Flexible Mapping items, accuracy feedback was provided, together with the highlighting of the correct answer option and a short explanation of the rule. This feature aimed at creating the foundation for solving the subsequent analogy within an item triplet and inhibiting the tendency to unnecessarily engage in inferring a new rule, which might be potentially triggered by the introduction of domain-heterogeneous stimuli.

#### 3.2.3. Conventional gf Measures as Reference Tests

Testing the Separability Assumption and the Predictive Utility Assumption necessitated the employment of “reference tests” of fluid intelligence measured using conventional approaches. The selection of such tests was informed by the objectives, to avoid a predominance of either a domain or item paradigm.

We chose two of the marker tests from the *French Kit of Factor-Referenced Cognitive Tests* (F-Kit: Ekstrom et al. 1976), the Letter Sets Test and the Locations Test. The third reference test was the Bongard Test from the Berlin Structure of Intelligence Test Battery (BIS, Jäger 1982, see also Bucik and Neubauer 1996). In the *Letter Sets Test* (LTR), five sets of four letters are presented. The task is to identify the rule which relates four of the sets to each other and to mark the one that is not in alignment with this rule. Performance in this test, which consists of 15 items, for which 7 min is allocated, is considered to be an indicator of levels of fluid intelligence. In the *Locations Test* (LOC), markings in four rows of places and gaps are presented. The task is to identify the rule that governs the locations of these markings and to mark the location on a fifth row accordingly. Performance in this test, which contains 14 items, for which 6 min is available, is also considered to be an indicator of fluid intelligence. In the *Bongard Test* (BON), in each of the 8 items, two sets of 6 graphic patterns each are presented. The task is to classify additional stimuli as belonging in one or the other set according to the identified rule that unites the patterns within each set but separates the patterns between the sets. Performance on this test, for which 6 min is available, is considered an indicator of fluid intelligence. Composite scores extracted from a Principal Component Analysis of these indicator tests composed a latent variable capturing fluid intelligence (*gf_comp*).

#### 3.2.4. GPA

Testing the Predictive Utility Assumption requires a criterion that is (a) meaningful, in terms of real-life relevance, and (b) reflective of the ability to deal with novelty in its different facets. In this context, we considered the transition from high school to college (university) as a process that requires flexibility. This transition is characterised by demands of dealing with ostensible novelty in terms of the challenges that the perceived newness and unfamiliarity of the social, geographical, and operational environment bring, even though similar situations have been successfully managed in the past. The transition from high school to university tends to also demand dealing with obscured novelty. This is in terms of the perceived familiarity with one of the primary tasks, i.e., to continue learning, yet the forms of engagement to do so effectively in this new environment are likely to differ from those that were experienced in high school. An operationalisation of the level of success in coping with these transition challenges can be found in students’ Grade Point Average (GPA). More specifically, the relative intra-individual consistency in participants’ GPA scores from high school and the first year of college constitutes a construct-relevant criterion to estimate the predictive utility of the newly developed test for cognitive flexibility.

## 4. Results and Discussion

Table 1 shows the descriptive statistics for the study variables, including their intercorrelations and the respective *N*s.

**Table 1:** Descriptive statistics for, and intercorrelations of, study variables (pairwise *N* in upper triangle).

	N	Mean	Stdev	FIT_typ	FIT_atyp1	FIT_atyp2	FIT_atyp_x	FMT_hom	FMT_het1	FMT_het2	FMT_het_x	LOC_gf	LTR_gf	BON_gf	gf_comp	HS_GPA	GPA_C1	congruent	incongruent
FIT_typ	271	.713	.142		271	271	271	223	223	223	223	223	236	235	224	230	217	223	223
FIT_atyp1	271	.546	.168	.51		271	271	223	223	223	223	223	236	235	224	230	217	223	223
FIT_atyp2	271	.432	.151	.34	.46		271	223	223	223	223	223	236	235	224	230	217	223	223
FIT_atyp_x	271	.489	.139	.50	.86	.85		223	223	223	223	223	236	235	224	230	217	223	223
FMT_hom	254	.651	.136	.42	.49	.42	.53		254	254	254	240	233	232	226	221	208	223	223
FMT_het1	254	.569	.153	.34	.34	.26	.35	.54		254	254	240	233	232	226	221	208	223	223
FMT_het2	254	.639	.168	.30	.34	.26	.35	.50	.55		254	240	233	232	226	221	208	223	223
FMT_het_x	254	.603	.142	.36	.39	.30	.40	.59	.87	.89		240	233	232	226	221	208	223	223
LOC_gf	256	.459	.197	.33	.35	.27	.37	.37	.44	.32	.43		249	248	241	223	210	221	221
LTR_gf	257	.755	.136	.35	.28	.23	.30	.28	.30	.22	.30	.36		252	241	216	203	220	220
BON_gf	253	.332	.242	.30	.32	.18	.30	.34	.26	.17	.24	.32	.30		241	215	202	219	219
gf_comp	242	.000	1.00	.44	.42	.31	.43	.44	.45	.32	.44	.76	.75	.72		210	197	213	213
HS_GPA	266	3.394	.457	.28	.25	.40	.37	.53	.46	.36	.46	.34	.35	.16	.38		246	190	190
GPA_C1	253	1.917	.588	.36	.36	.44	.46	.60	.51	.42	.53	.37	.28	.28	.42	.74		177	177
congruent	223	.692	.977	.84	.59	.45	.62	.84	.52	.47	.57	.42	.37	.38	.53	.48	.57		223
incongruent	223	.558	.966	.51	.74	.68	.83	.67	.74	.75	.84	.48	.35	.32	.52	.50	.59	.71	

Notes: FIT: Flexible Inference Task; FMT: Flexible Mapping Task; FIT_typ: typical; FIT_atyp1: first atypical; FIT_atyp2: second atypical; FIT_atyp_x: combined first and second atypical; FMT_hom: homogeneous; FMT_het1: first heterogeneous; FMT_het2: second heterogeneous; FMT_het_x: combined first and second heterogeneous; LOC_gf: Locations Test; LTR_gf: Letter Sets Test; BON_gf: Bongard Test; gf_comp: Gf composite score; HS_GPA: high school GPA; GPA_C1: first-year GPA (This variable has been rescaled to adjust for differences in selectivity of universities involved.); Congruent: combined typical and homogeneous; Incongruent: combined atypical and heterogeneous. See Materials and Method section for details on derivation.

### 4.1. Complexity Assumption

The first assumption tested in this exploratory proof-of-concept study referred to the effects of the systematic complexity manipulation on difficulty when being confronted with domain-atypical classification items (Flexible Inference Task) or domain-heterogeneous analogy items (Flexible Mapping Task), respectively, in comparison to their domain-typical or domain-homogeneous counterparts (Complexity Assumption).

**Figure 5:**
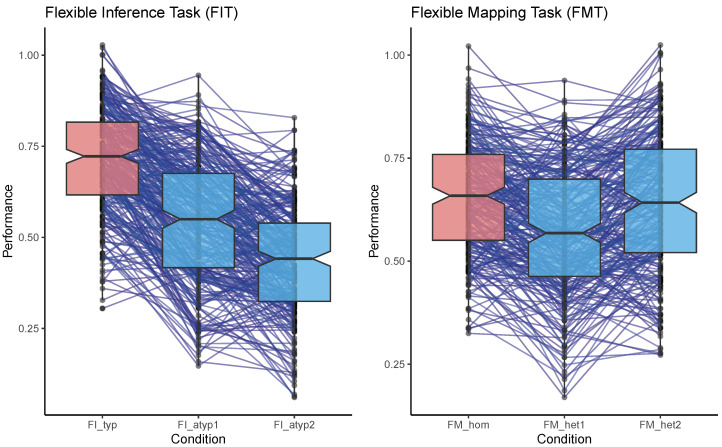
Difficulty differences between domain-typical and domain-atypical (FIT) and domain-homogeneous and domain-heterogeneous (FMT) versions of items. Lines link individuals’ performances across item triplets.

The results of a repeated measurement ANOVA for performance scores in the Flexible Inference Task (left panel in Figure 5) revealed that domain-atypical classification items introduced the demand to deal with obscured novelty, which resulted in a statistically significant performance drop across items within the triplet (*F*_2,540_ = 421.423, *p* < .001, generalised η^2^ = 0.36). Post hoc analyses with a Bonferroni adjustment indicated that all the pairwise comparisons between items within a triplet showed statistically significant drops in performance (typical vs. atypical 1: *t*_270_ = 17.4, *d* = 1.06; atypical 1 vs. atypical 2: *t*_270_ = 11.9, *d* = 0.72; typical vs. atypical 2: *t*_270_ = 28.0, *d* = 1.70).

The same analyses conducted for the Flexible Mapping Task (right panel in Figure 5) also indicated a general effect of the introduction of the demand to deal with ostensible novelty caused by domain-heterogeneous analogy items (*F*_2,506_ = 48.097, *p* < .001, generalised η^2^ = 0.05). The post hoc analyses help to contextualise the fact that this effect was considerably smaller in comparison to the Flexible Inference Task. Whereas the transition from domain-homogeneous to domain-heterogeneous analogy items resulted in the expected drop in performance (homogeneous vs. heterogeneous 1: *t*_253_ = 9.85, *d* = 0.62), the confrontation with a domain-heterogeneous version of the same item after its other domain-heterogeneous version resulted in an improvement (heterogeneous 1 vs. heterogeneous 2: *t*_253_ = −7.63, *d* = −0.48). This pattern suggests a recovery from the initially incurred domain-switching costs, so much so that performances on the second domain-heterogeneous items within each triplet tended to be on the same level as performances on their respective domain-homogeneous counterpart (homogeneous vs. heterogeneous 2: *t*_253_ = 1.24, *d* = 0.09, *p* > .216). Success in dealing with ostensible novelty is based on the realisation that the rule inferred for the domain-homogeneous version of an item also applies to its domain-heterogeneous versions.

The results of testing the *Complexity Assumption* support the expectation that adding the demand for inhibition to the sequence of cognitive information-processing steps—realised through domain-atypical classification items in the Flexible Inference Task, or domain-heterogeneous analogy items in the Flexible Mapping Task—is reflected in systematic differences in performance.

### 4.2. Separability Assumption

**Figure 6:**
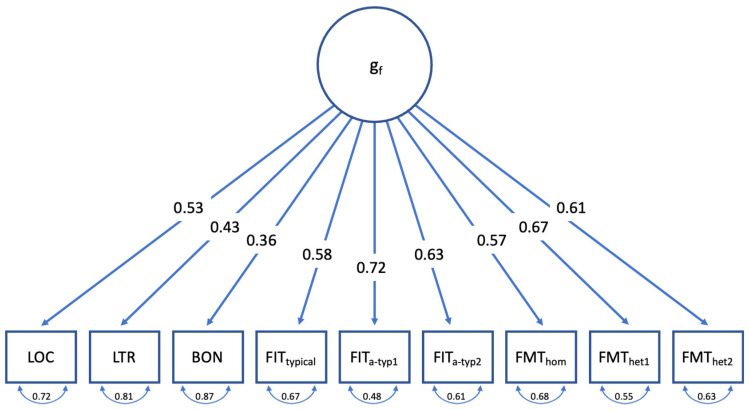
Single-factor gf model.

**Figure 7:**
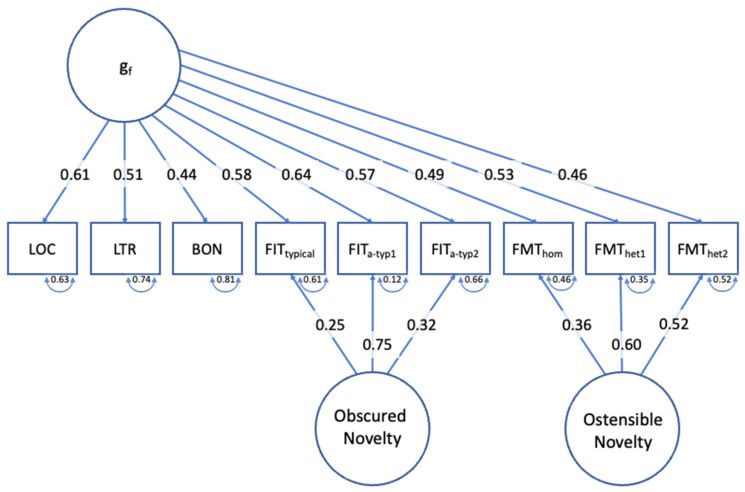
Bi-factor model.

The second assumption tested in this exploratory proof-of-concept study referred to the question of whether the newly developed flexibility tasks—whilst contributing to the positive manifold that constitutes a general factor of cognitive abilities—captured substantial systematic variance that is not reflected in conventional tests of fluid intelligence. To test the Separability Assumption, we subjected the performance data to a Confirmatory Factor Analysis and compared the fit of a simple (and parsimonious) general-factor model (Figure 6) with the fit of a bi-factor model, in which performances in the Flexible Inference Task and Flexible Mapping Task were modelled as an additive combination of the Gf-factor and a respective flexibility factor (Figure 7).

The single-factor model (Figure 6) produced a not too discouraging, yet suboptimal, fit (χ^2^_27_ = 92.048, *p* < .001, CFI = .867, TLI = .823, RMSEA = .105). The relative homogeneity of factor loadings indicates that the flexibility tasks also contributed to the positive manifold constituting the g-factor in this model. This result is to be expected, given that the newly developed flexibility tasks made use of the same item paradigms that can be found in conventional measures of *gf* (e.g., classification and analogies).

The bi-factor model (Figure 7) showed a significantly better fit (χ^2^_21_ = 23.19, *p* = .33, CFI = .996, TLI = .992, RMSEA = .022). The differences in fit statistics between the single-factor model and the bi-factor model are presented in Table 2. This result lends support for the viability of the Separability Assumption, which asserted that the newly developed flexibility tasks evoke a systematic variance in performance that is in addition to what these tests share in terms of their contribution to a general factor. These two task-specific special factors cannot be interpreted as domain-specific factors, as the domain was completely counterbalanced within and between the two flexibility tasks.

### 4.3. Predictive Utility Assumption

The Predictive Utility Assumption was built on the expectation that, despite using conventional item paradigms (i.e., classification problems and analogy problems, respectively), performance on *domain-atypical* classification items (Flexible Inference Task) and on *domain-heterogeneous* analogy items (Flexible Mapping Task) added incrementally to the prediction of the success with which the transition from high school to university was mastered academically. To test this assumption, participants’ *first-year college GPA* was regressed onto flexibility scores, whilst controlling for (traditionally operationalised) general cognitive abilities (*gf_comp*) and pre-university GPA (HS_GPA). Table 3 and Table 4 present the results of the regression analyses conducted for the Flexible Inference Task and the Flexible Mapping Task, separately.

Table 5 shows the estimates of a regression, for which performances in the domain-typical classification items (FIT-typical) and the domain-homogeneous analogies (FMT-homogeneous) were combined in a predictor labelled “congruent”, and performances in the domain-atypical classification items (FIT-atypical) and the domain-heterogeneous analogies (FMT-heterogeneous) were combined in a predictor labelled “incongruent”.

The outcomes of the task-specific analyses (Table 3 and Table 4), as well as the combined analyses (Table 5), consistently indicate that the experimental variation of item presentation conditions in the newly developed flexibility tasks was able to contribute predictive value beyond conventional measures of *g_f_*, supporting the Predictive Utility Assumption.

## 5. General Discussion

Navigating changing demands in a dynamic world requires adaptive contingent behaviour. The concept ‘flexibility’ can be used as a descriptive label for such behaviour. Whilst flexibility in behaviour depends on a wide range of person characteristics, including knowledge, experience, affect, motivation, and other so-called non-intellectual attributes, the present study focuses on the abilities to perceive, to encode, and to process information related to the problem or situation encountered. This narrows down the perspective from flexibility as a meta-competency (Yu et al. 2019) to cognitive flexibility as a cognitive ability construct. It also recommends theories of information processing to be used as a conceptual framework.

### 5.1. What We Did

In this article, we started by contemplating whether cognitive flexibility requires the “new construct” treatment. Consultation of the extant literature suggests that the notion of cognitive flexibility has always been an essential element of our understanding of intelligence in general, and of fluid intelligence in particular. In other words, definitions of intelligence of almost any couleur or tradition comprise references to dynamic aspects of cognitive functioning, including the ability to adapt to changes and to deal with novelty. In this regard, there seems no need for conceptual inventiveness in propagating a new construct. What is apparent, however, is that conventional approaches to measuring fluid intelligence routinely fail to provide opportunities for test takers to exhibit behaviour that can be considered indicative of an ability to deal with novelty. As a result, conventional operationalisations of fluid intelligence insufficiently represent manifestations of cognitive flexibility as an ability construct. To mitigate this issue of construct underrepresentation (e.g., Messick 1995), operationalisation and conceptualisation need to be better aligned. If anything, such an alignment requires innovativeness in terms of operationalisation, rather than conceptual inventiveness.

With the present article, we address the misalignment of conceptualisation and operationalisation. We start by conceptualising cognitive flexibility as an emerging property of a set of information-processing components required to adapt to contingencies caused by changes in situational or task demands or in characteristics of persons. Such changes, or deviation from “the usual”—once perceived as such by the problem solver—induce the impression of novelty, which can take two distinct forms. These two forms are obscured novelty and ostensible novelty. Obscured novelty emerges when surface features of a problem situation create a false sense of familiarity, which then might encourage the deployment of a known, but, in this situation, inadequate, problem-solving approach. Ostensible novelty emerges when surface features of a problem situation trigger a false sense of novelty, which subsequently tends to result in unnecessary engagement in a search for a novel approach to solving the problem at hand, whilst an already-learned approach would suffice. In terms of cognitive processing components, dealing with obscured novelty requires an inhibition of previously employed approaches to solve a problem, or disengagement from information that is no longer relevant in the given novel context. Dealing with ostensible novelty requires one to resist the tendency to falsely inhibit the employment of a known, familiar, or previously successfully utilised approach and to engage in unnecessary cognitive processing steps instead.

To measure the ability to deal with obscured novelty and ostensible novelty, respectively, we have developed the Flexible Inference Task and the Flexible Mapping Task. The Flexible Inference Task is composed of classification items and the Flexible Mapping Task is composed of analogy items, both using numbers, geometric shapes, and words as stimuli. To evoke the demand of dealing with either form of novelty, items in these tasks are presented in two versions, a domain-congruent version and a so-called domain-incongruent version. Domain-congruent items resemble those typically found in classification problems or analogy problems, as they are used in psychometric tests of fluid intelligence. In the Flexible Inference Task, solving domain-congruent items relies on inferring classification rules based on domain-typical features of the stimuli (e.g., the meaning of words, arithmetic operations with numbers). As is standard in conventional approaches to measuring fluid intelligence, solving the domain-congruent analogy items in the Flexible Mapping Task requires one to infer relationships between elements that are all stemming from the same domain (i.e., words, numbers, shapes).

To capture the within-person processes of dealing with the two facets of novelty as described above, items in both tasks are also presented in a domain-incongruent version. For the respective items in the Flexible Inference Task, this form of presentation means that the to-be-inferred classification rule is based on domain-atypical features of the stimuli used (e.g., number of syllables in words, the shape of numbers). The inclusion of these items creates the demand for dealing with obscured novelty. For the Flexible Mapping Task, the demand for dealing with ostensible novelty is induced by presenting items in which the relationship inferred between the two stimuli in the analogy stem needs to be mapped onto stimuli from a different domain (e.g., the inferred relationship between two words based on their meaning is to be mapped onto numbers). The systematic variation of the characteristics of the stimulus material across otherwise identical items aims at evoking intra-individual variability in performance, which we assert to be indicative of cognitive flexibility.

### 5.2. What We Have Found

We tested three assumptions: The *Complexity Assumption* posits that the differences in complexity between congruent and incongruent items will be reflected in systematic differences in the difficulty problem solvers experience. The *Separability Assumption* posits that these complexity differences represent psychometrically differentiable effects, rather than mere differences in levels of difficulty. The *Predictive Utility Assumption* posits that these newly developed flexibility tasks show incremental value in predicting success in the transition from high school to university as an indicator of the ability to adjust to changes and to deal with novelty. Together, they contribute to a test of the conceptual coherence of an account of cognitive flexibility.

Our conceptualisation of cognitive flexibility as an emerging quality of a sequence of cognitive processing steps lays the ontological foundation for exploring its measurability in the context of a reflective measurement model. The analyses and results help us address the epistemological question of whether the newly developed tasks are valid measures of cognitive flexibility, by establishing whether performance scores are causally affected by variation in this attribute. The theory of response behaviour to be tested for this purpose is anchored in an information-processing paradigm. The results related to both testing the Complexity Assumption and the Separability Assumption corroborate the notion of a construct-adequate measurement of cognitive flexibility. The results related to testing the Predictive Utility Assumption help to give meaning to the observed statistical association between the performance scores obtained in these flexibility tasks and the quantification of students’ academic performance^3^.

### 5.3. Cognitive Flexibility: A Tentative Précis

Cognitive flexibility, defined as an ability construct, represents one of the conceptual core components of fluid intelligence. However, despite this, cognitive flexibility tends to be underrepresented in conventional operationalisations of fluid intelligence. As a dynamic ability construct, cognitive flexibility refers to individual differences in the within-person cognitive processes for dealing with novelty, as they are involved in successfully adapting to changing demands in a dynamic world. Cognitive flexibility enables individuals to functionally adjust their behaviour contingent on the task, its demands, and the situational dynamics of the environment, concordant with their capabilities to respond.

### 5.4. Where to from Here?

The alignment of conceptualisation and operationalisation as the main objective of this research is achieved by adopting a methodological approach that more appropriately mirrors the dynamic aspects of behaviour organisation relevant when tackling challenges that require functional responses to change. Such an approach entails systematic variations of item characteristics (e.g., domain-congruency vs. domain-incongruency), which constitute repeated within-person experiments. This approach allows for causal inferences regarding the relationship between variations in the to-be-measured attribute (i.e., cognitive flexibility) and the score variations observed between (or within) individuals. Establishing such a causal relationship represents the necessary precondition for meaningful claims of validity (Borsboom et al. 2004; Markus and Borsboom 2013).

It needs to be acknowledged that the scoring approach employed for both tasks in the proof-of-concept study reported here is rather simplistic, i.e., accuracy in form of the relative number of correct responses across items. Admittedly, this form of scoring falls far short of the conceptually implied potential, which would, for example, stipulate to focus on intra-triplet variability, be it in terms of accuracy, response latencies (Beckmann and Beckmann 2005), or a combination of both. The main reason for retaining the simplest approach to scoring was to ensure that the outcomes of the assumption testing could be attributed to the conceptually informed changes in task presentation, rather than to the employment of a more sophisticated psychometric scaling and modelling approach. Whilst this might be appropriate for a proof-of-concept study such as the one presented here, future research is expected to work towards an even closer alignment of the operationalisation with a conceptualisation of fluid intelligence that incorporates cognitive flexibility in the form of intra-individual variability in contingent adaptive behaviour.

The methodological approach of systematically manipulating variations of the task and/or the situational characteristics of test items according to theory, to evoke a predicted intra-individual variability in test performance, is not particularly novel. It is, in fact, at the core of the psychometric assessment of learning ability or intellectual change potential (Beckmann 2014; also, see Guthke and Wiedl 1996; Guthke and Beckmann 2000b), where it has been extensively discussed, researched, and successfully employed through so-called Dynamic Testing^4^. We therefore see potential for future research into the development of theories and subsequently assessment tools to benefit from insights gained related to the paradigm of Dynamic Testing.

For prescriptions of actions to be meaningful, for instance, in relation to future research directions or devising interventions, one must go beyond a mere description of the lamentable status quo (e.g., simply stating there is a misalignment between conceptualisation and operationalisation). Prescriptions need to be informed and underpinned by explanations. The following reflections should be seen as an attempt to further our understanding not only of the reasons for the emergence of this misalignment but also of the mechanisms that are likely to have contributed to its preservation. In the following sections, we will briefly outline two possible reasons we believe deserve greater attention in future research. One such reason refers to the importance of building the operationalisation of cognitive flexibility on a reflective measurement model. The other potential reason refers to the necessity of aligning measurement and analysis models with the within-person perspective stipulated conceptually by the definition of cognitive flexibility.

#### 5.4.1. Models of Measurement

One factor contributing to the misalignment between conceptualisation and operationalisation may lie in an insufficient differentiation between formative measurement models and reflective measurement models (Van der Maas et al. 2014) when it comes to attempts to measure constructs such as cognitive flexibility (Birney and Beckmann 2022). Indices derived in the context of formative measurement models represent a form of ‘condensation’ of the information contained in performance scores obtained across a number of items (or tests). This process results in a composite score. If the main objective were to predict some kind of behaviour or other quantifiable outcome (e.g., success in academic endeavours), reliance on a composite score derived from a formative measurement model might suffice. The dependence of the composite score on the composition of the item set that underpins it allows the levels of perceived precision of prediction to be optimised through more or less pragmatically informed item selections (e.g., Steger et al. 2023) or according to some psychometric homogeneity constraints. A consequently maximised correlation between the composite score and the variable that serves as a criterion does not, however, indicate validity in terms of having measured cognitive flexibility as an ability construct. The other implication of a formative measurement model is that a construct like cognitive flexibility, as defined here, is unlikely to emerge or “to be formed” from a collection of static tasks. This again, however, does not mean that cognitive flexibility does not exist, or that it does not matter. The employment of reflective measurement models builds on a commitment to ontological realism (i.e., stipulating that cognitive flexibility as a latent construct exists independently of attempts to measure it), which in turn relies on ex ante conceptualisations of the targeted attribute, including causal models of intra-individual variation in response behaviour. The epistemological problem is easily obfuscated by the fact that performance data as such—be it in the context of primary or secondary data analyses—do not necessarily reveal whether these data are a result of a formative quantification or a result of a reflective measurement process. Future research aimed at establishing validity of measurement and theory testing can only be addressed based on reflective measurement models.

Using GPA as a criterion for incremental utility—as done in the present study—is not without problems. School grades, or any other form of quantified proxies of academic proficiency, are somewhat ‘low hanging fruits’ (Sternberg 2019, p. 7) in the context of seeking empirical evidence for the usefulness of test scores. Also, success in tackling scholastic challenges builds on more than reasoning ability, regardless of whether it is measured statically (c.f., conventional approaches to ability testing) or dynamically, as done in the present study. It stands to reason that success in mastering the transition from high school to university also benefits from social skills and many other so-called non-cognitive attributes, which, arguably, are not reflected in the performance scores obtained in the flexibility tasks introduced here. Looking at this issue from a slightly different angle, the strength of the association between scores on the flexibility tasks used here and students’ first-year college GPA tells us more about the criterion (e.g., the role cognition plays in forming adaptive contingent behaviour) than the chosen predictors themselves. To reiterate, such correlations are not to be misunderstood or misrepresented as validity indicators of the tasks. Validity-focused research needs to be based on reflective measurement models and needs to focus on testing causal hypotheses linking the conceptual (i.e., theory) and the empirical (i.e., performance), conducted in the context of experimental research.

#### 5.4.2. Ergodicity

Another potential reason for the misalignment between the conceptualisation and operationalisation of cognitive abilities has already been mentioned. Largely underrepresented in conceptual and subsequent methodological considerations, intelligence conceived as cognitive flexibility (Birney and Beckmann 2022) is primarily a within-person phenomenon. Conventional operationalisations of cognitive abilities, however, seem primarily dominated by between-person accounts. As Molenaar (e.g., Molenaar 2004a, 2004b, 2013; Molenaar and Campbell 2009) and others (Schmiedek et al. 2020) keep reminding us, the assumption that results obtained from analyses of inter-individual variation are validly generalisable to an intra-individual level is untenable (i.e., an ergodicity problem). The occurrence of change in the situational characteristics of a task is expected to cause changes in behaviour, which in psychological measurement is operationalised in the form of performance scores. The effects of such changes vary in strength between individuals. They might also differ systematically within individuals. A construct-adequate operationalisation of cognitive flexibility therefore needs to be based on within-person modelling (Birney et al. 2019). In other words, future research needs to further refine reflective measurement models and analysis approaches, so that performance scores are verifiably valid operationalisations of conceptually conceived inter-individual differences in intra-individual variability. Progress in theory development and assessment practice will be affected by how effectively the measurement model issue and the ergodicity issues are addressed.

Analogously to dealing with the challenges of obscured and ostensible novelty, the points raised in this paper have the potential to invite a *jingle-jangle* fallacy^5^. Falsely assuming that cognitive flexibility is a new construct would be an example of a *jangle* fallacy; erroneously assuming that cognitive flexibility (as a facet of fluid intelligence) is “covered” by conventional assessment approaches (i.e., psychometric *gf* tests) would be an example of a *jingle* fallacy. We hope, however, to have contributed to better navigating the jingle-jangle jungle by highlighting that it is the discrepancy between conceptualisation and operationalisation that has held back the development of valid measures. We also present a possible solution to the misalignment problem, which at least offers an orientation for a way forward methodologically.

## Figures and Tables

**Table 2:** Comparison of fit statistics.

Model	*df*	AIC	BIC	χ^2^	Δχ^2^	RMSEA	*df*	*p*
bi-factor model	21	−2082.2	−2001.0	23.186				
gf-factor model	27	−2025.4	−1964.5	92.048	68.862	.21923	6	<.0001

**Table 3:** Flexible Inference Task—regression results using first-year college GPA as criterion, controlling for last-year high school GPA and gf [*N* = 172].

Predictor	*b*	*b* CI_95%_	β	β CI_95%_	Fit	Difference
(Intercept)	−2.80 **	[−3.77, −1.84]				
HS_GPA	1.30 **	[1.04, 1.55]	0.62	[0.50, 0.74]		
gf_comp	0.09	[−0.04, 0.23]	0.09	[−0.04, 0.22]		
FIT_typ	0.62	[−0.20, 1.44]	0.09	[−0.03, 0.21]		
					*R*^2^ = .501 **	
					CI_95%_ [.39, .58]	
(Intercept)	−2.83 **	[−3.77, −1.89]				
HS_GPA	1.21 **	[0.96, 1.46]	0.58	[0.46, 0.70]		
gf_comp	0.06	[−0.07, 0.19]	0.06	[−0.07, 0.19]		
FIT_typ	0.10	[−0.77, 0.98]	0.02	[−0.12, 0.15]		
FIT_atyp	1.40 **	[0.44, 2.35]	0.20	[0.06, 0.33]		
					*R*^2^ = .525 **	Δ*R*^2^ = .015 *
					CI_95%_ [.41, .60]	CI_95%_ [.01, .06]

Note: * *p* < .01, ** *p* < .001.

**Table 4:** Flexible Mapping Task—regression results using first-year college GPA as criterion, controlling for last-year high school GPA and gf [*N* = 176].

Predictor	*b*	*b* CI_95%_	β	β CI_95%_	Fit	Difference
(Intercept)	−3.05 **	[−3.84, −2.25]				
HS_GPA	1.11 **	[0.86, 1.36]	0.53	[0.41, 0.65]		
gf_comp	0.09	[−0.03, 0.20]	0.09	[−0.03, 0.20]		
FMT_hom	2.01 **	[1.15, 2.87]	0.28	[0.16, 0.40]		
					*R*^2^ = .592 **	
					CI_95%_ [.49, .66]	
(Intercept)	−3.19 **	[−3.99, −2.40]				
HS_GPA	1.05 **	[0.80, 1.30]	0.50	[0.38, 0.62]		
gf_comp	0.05	[−0.06, 0.17]	0.05	[−0.06, 0.17]		
FMT_hom	1.51 **	[0.58, 2.44]	0.21	[0.08, 0.34]		
FMT_het	1.15 *	[0.25, 2.04]	0.17	[0.04, 0.30]		
					*R*^2^ = .606 **	Δ*R*^2^ = .015 *
					CI_95%_ [.51, 0.67]	CI_95%_ [0.01, 0.04]

Note: * *p* < .01, ** *p* < .001.

**Table 5:** Flexible Inference Task and Flexible Mapping Task combined—regression results using first-year college GPA as criterion, controlling for last-year high school GPA and gf [*N* = 163].

Predictor	*b*	*b* CI_95%_	β	β CI_95%_	Fit	Difference
(Intercept)	−3.61 **	[−4.56, −2.67]				
HS_GPA	1.21 **	[0.95, 1.46]	0.56	[0.45, 0.68]		
gf_comp	0.06	[−0.07, 0.19]	0.06	[−0.07, 0.18]		
congruent	2.25 **	[1.14, 3.35]	0.26	[0.13, 0.39]		
					*R*^2^ = .570 **	
					CI_95%_ [.47, .64]	
(Intercept)	−3.65 **	[−4.56, −2.74]				
HS_GPA	1.10 **	[0.85, 1.35]	0.51	[0.40, 0.63]		
gf_comp	0.02	[−0.11, 0.15]	0.02	[−0.10, 0.14]		
congruent	1.09	[−0.16, 2.35]	0.13	[−0.02, 0.27]		
incongruent	2.17 **	[0.93, 3.42]	0.26	[0.11, 0.40]		
					*R*^2^ = .601 **	Δ*R*^2^ = .030 *
					CI_95%_ [.50, 0.66]	CI_95%_ [.00, .06]

Note: * *p* < .01, ** *p* < .001.

## Data Availability

The data presented in this study are available at [https://osf.io/f7t3b/?view_only=0d3500f88fff40ffab75cd2aa2f4d317]. Accessed on 18 November 2023.

## Notes

1 Interestingly, the notion of progressive adaptability suggests conceptual overlap with the ability to learn, which is another aspect of intellectual functioning that is insufficiently represented in conventional approaches to the measurement of intelligence (Beckmann 2006, 2014).

2 Please note that “domain” refers to the stimuli in the analogy stem, i.e., these are the terms A and B in the analogy of the form A : B :: C : ??.

3 As has been extensively discussed elsewhere (e.g., Borsboom et al. 2004; Borsboom 2005), it is important to realise that correlations between task performance and some criterion cannot be interpreted as indicators of some form of validity (e.g., “predictive validity”).

4 Note, the term ‘Dynamic Testing’ is not to be confused with the term ‘Dynamic Assessment’ (for clarification, see e.g., Guthke and Beckmann 2000a). One of the main differentiators is that the term ‘Dynamic Testing’ refers to the psychometric approach to measuring intellectual change potential (assessment via response to intervention), whilst the term ‘Dynamic Assessment’ (Feuerstein et al. 1979) tends to refer to a clinical approach with a primarily intervention focus (i.e., assessment as monitoring progress in interactively mediated learning).

5 The obscured novelty induced in the Flexible Inference Task invites a jingle fallacy; the ostensible novelty induced in the Flexible Mapping Task invites a jangle fallacy.

## Appendix A

Solutions for the example items used in this paper:

*Figure 1:*

FIT_words_: top left (rhyme); FIT_numbers_: top right (11 × 9 = 99); FIT_shapes_: bottom right (rectangular)

*Figure 2:*

FIT_words-left_: top right (palindrome); FIT_words-right_: bottom right (3 letters);
FIT_numbers-left_: bottom left (symmetry); FIT_numbers-right_: bottom left (cross sum = 18);
FIT_shapes-left_: bottom left (vertical); FIT_shapes-right_: top left (number of features = 6);

*Figure 3:*

FMT_words_: top right (synonyms); FMT_numbers_: bottom left (quadrupling); FMT_shapes_: bottom right (two thirds)

*Figure 4:*

FMT_words-shape_: bottom left (equivalent, same numbers of squares); FMT_words-numbers_: top right (equivalent);
FMT_numbers-words_: bottom right (quadrupling); FMT_numbers-shapes_: top right (quadrupling);
FMT_shapes-words_: top right (two thirds); FMT_shapes-numbers_: top right (two thirds).
